# A dataset of shallow soil moisture for alfalfa in the Ningxia irrigation area of the Yellow River

**DOI:** 10.3389/fpls.2024.1472930

**Published:** 2024-11-28

**Authors:** Yongqi Ge, Jing Wang, Rui Liu, Lifeng Lu, Songtao Yang, Daotong Tang, Ang Li, Zixin Zhu

**Affiliations:** College of Information Engineering, Ningxia University, Yinchuan, China

**Keywords:** alfalfa, shallow soil moisture, water amount, meteorological data, deep learning

## 1 Introduction

Alfalfa is the most widely planted perennial forage crop in the world, serving as a vital supplementary feed for cattle and requiring substantial water inputs (Zhang et al., 2023; Fink et al., 2022; Jia et al., 2024). Precision field water management can improve alfalfa yield without necessitating increased use of fertilizers or insecticides (Bai et al., 2022; Kayatz et al., 2024). However, challenges such as global warming, drought, and excessive water use have intensified water scarcity (Rosa et al., 2018; Salehi, 2022; Gerten et al., 2020), particularly in the Ningxia Irrigation Area of the Yellow River (NIR) in northwest China. This region, characterized by arid conditions and minimal rainfall, heavily relies on the Yellow River for irrigation. Yet, the effective utilization rate of this water source remains relatively low (Zhang et al., 2022). Currently, alfalfa irrigation in the NIR follows quota schedules based on historical field experiences, lacking adaptability to fluctuating precipitation and soil moisture dynamics, which leads to inefficient water use (Du et al., 2020; Zhou et al., 2019). Therefore, improving water management efficiency for alfalfa in the NIR is crucial for promoting regional cultivation and optimizing water resource utilization.

Predicting soil moisture at multiple depths within alfalfa’s root zone is crucial for the early detection of drought or over-irrigation, mitigating trends in soil salinization, and enhancing the assessment of water and nutrient availability in areas with concentrated alfalfa roots. Current research extensively employs soil moisture sensors to monitor the dynamics of soil moisture and assess crop growth comprehensively (Hu et al., 2019). For example, Yan et al. (2019) conducted experiments involving five water levels and rain-fed plots to compare the growth of alfalfa under various irrigation conditions. Sun et al. (2022) analyzed soil water content, salinity, and temperature across different fertilizer levels to study their impact on soil moisture and seedling emergence rates. However, high-precision multi-depth soil moisture sensors are typically costly and may lack adequate predictive capability.

Additionally, remote sensing technology and radar facilitate the rapid detection of soil moisture. Zhu et al. (2021) employed remote sensing to gather soil moisture data from 14 stations in the 0-5 cm layer, covering the period from April 2017 to October 2019. They applied the inverse distance interpolation algorithm to predict soil moisture at the station scale. Some studies integrate neural networks with multi-source remote sensing data to estimate soil moisture using inversion models (Jia et al., 2021), while others have combined satellite data with soil texture, terrain, and climate variables for prediction (Celik et al., 2022). However, the collection and processing of remote sensing and radar data can be costly and are susceptible to weather conditions and surface vegetation cover, which limits their effectiveness for precise monitoring of soil moisture at multiple depths in small-scale farmland (Weihermüller et al., 2007; Guo et al., 2021).

With advancements in artificial intelligence, researchers are increasingly adopting deep learning models to simulate and predict soil moisture. Han et al. (2021) developed a model focused on forecasting soil moisture for the subsequent six days. Yu et al. (2020) utilized continuous meteorological and soil moisture data, integrating ResNet and BiLSTM to extract high-dimensional spatial and temporal features, thereby achieving accurate predictions of soil moisture. Recently, Liu et al. (2024) proposed a dual-branch combined deep learning model for predicting multi-depth soil moisture in alfalfa using time series data, demonstrating improved prediction accuracy, particularly under conditions of instantaneous water supplementation. These studies highlight the effectiveness of deep learning models in leveraging historical soil moisture data to predict moisture at various depths. Their robust data fitting and generalization capabilities enhance prediction accuracy and reduce agricultural input costs. However, predictive performance is heavily dependent on the quality and quantity of available data from the study area.

At present, numerous studies are concentrating on soil moisture datasets, emphasizing spatiotemporal variations in soil moisture and its correlation with soil properties (Mälicke et al., 2020; Martínez-Fernández et al., 2021). In the atmospheric dynamics-alfalfa-soil system, soil moisture varies due to meteorological conditions, crop growth, soil basal, and field management practices. Alfalfa, as a perennial crop, impacts soil moisture differently across various planting years. Moreover, in arid and semi-arid regions, limited rainfall and varying precipitation intensities influence soil moisture levels at different depths. Obtaining high-quality datasets that reflect diverse water replenishment scenarios necessitates extensive, long-term field experiments conducted across various depths and conditions. However, publicly available datasets specific to this system, particularly those that include shallow multi-depth soil moisture data related to alfalfa growth and environmental factors, are scarce.

In light of this context, this study established a comprehensive dataset of soil moisture and its spatiotemporal variations at shallow depths for alfalfa in the NIR. The dataset integrates meteorological data, shallow soil moisture measurements (0-10 cm, 10-20 cm, and 20-30 cm), alfalfa growth metrics, and field management practices. The aim of this study is to reduce agricultural input costs and provide data support for optimizing field water management methods for alfalfa in the NIR.

## 2 Value of the data

(1) This study established a shallow soil moisture dataset for alfalfa, encompassing different precipitation years, irrigation methods, and water and nitrogen levels. The dataset includes data from 12 cuts of alfalfa in 2017, 2018, 2022, and 2023, totaling 139,213 shallow soil moisture data points (31,206 in 2017, 36,339 in 2018, 50,868 in 2022, and 20,800 in 2023). It addresses the gap in publicly available shallow soil moisture data for alfalfa in the northwest agricultural-pastoral transitional zone. This dataset serves as a reliable source for developing deep learning models to accurately predict soil moisture at multiple depths for alfalfa, thereby supporting the optimization of field water management in the NIR.

(2) This dataset includes meteorological data, crop growth metrics (such as leaf area index, growth periods, and yield), soil temperature records, and field management details across various planting years and cuts of alfalfa. Specifically, it comprises 49,358 meteorological records, 695 entries for alfalfa leaf area index, 46 growth period records, 37 yield measurements, and 81,564 soil temperature readings. The integration of these diverse features enhances the dataset’s utility for multi-faceted modeling, thereby improving the predictive performance of models.

## 3 Materials and methods

### 3.1 Study area

**Figure 1:**
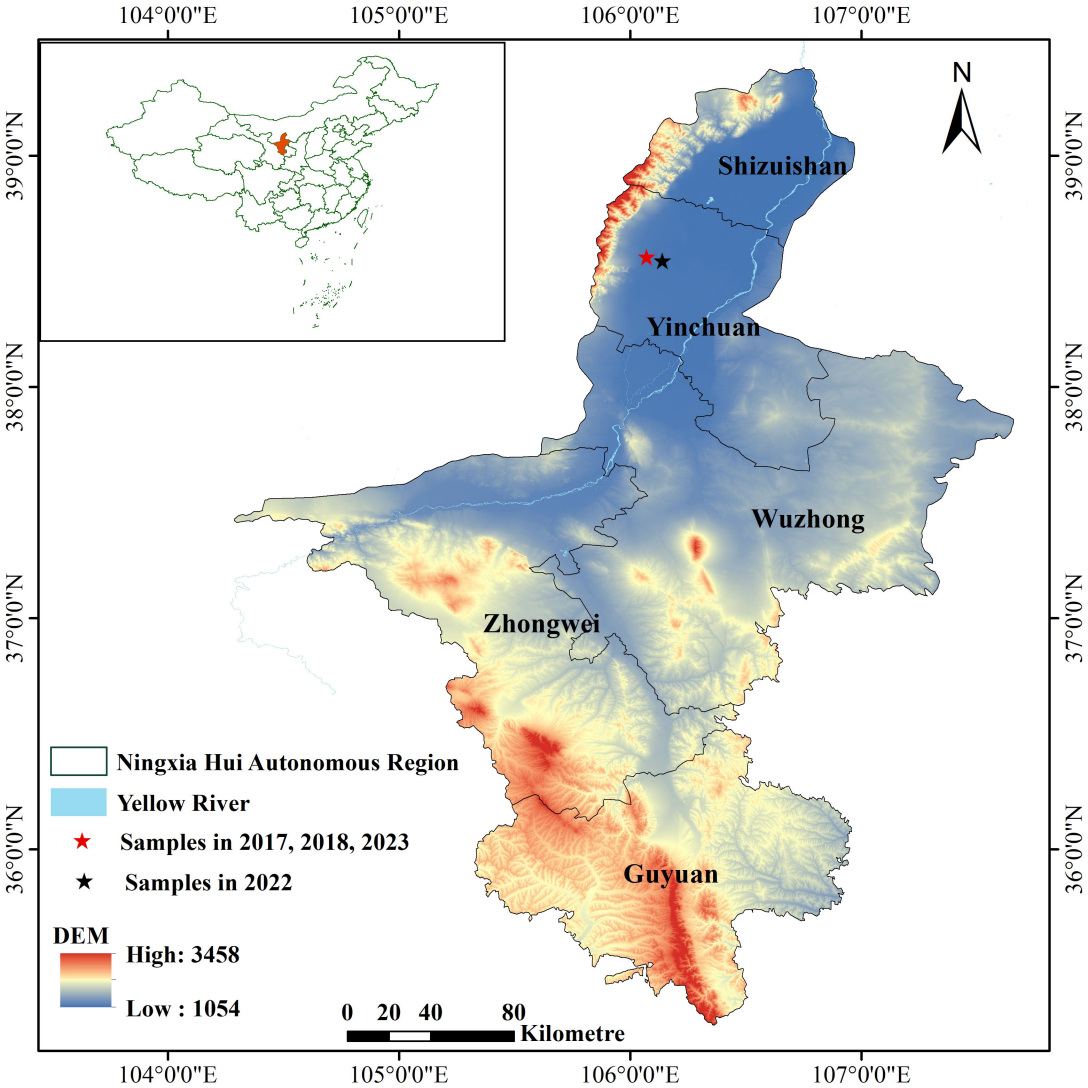
Location of the study area and spatial distribution of meteorological and soil sampling points.

In this study, the interest area is NIR, located in the Ningxia Hui Autonomous Region in China, characterized by a temperate continental climate. The NIR extends from 37.4° to 39.6° North and from 104.9° to 106.8° East. **Figure 1** shows the distribution of the monitoring stations within the study area.

The experimental area is situated at the eastern foot of the Helan Mountain within the Helan Mountain alluvial fan plain. The average altitude is 1120 m. The mean annual temperature is 8.4°C. The mean annual sunshine duration ranges from 2800 to 3000 hours, and the mean annual evaporation is approximately 3000 mm. The annual precipitation ranges from 180 to 200 mm, primarily concentrated from June to September. The soil texture is classified as light loam soil, specifically light gray calcium soil. The soil composition consists of approximately 58.39% sand particles, 15.86% silt particles, and 25.76% clay particles. The pH value of the 0-30 cm soil layer is 8.61, with an organic matter mass fraction of 13.4 g/kg, a total nitrogen mass fraction of 0.76 g/kg, an available phosphorus mass fraction of 10.65 mg/kg, and an available potassium mass fraction of 128.26 mg/kg. The soil basal characteristics of the experimental area are shown in **Table 1**. We employed the Wilcox method to measure field capacity at various depths. The cutting-ring method was used to assess saturated capacity, while the oven-drying method was applied to determine bulk density. Additionally, the traditional biological method was used to evaluate the wilting point.

**Table 1:** The soil basal characteristics of experimental area.

Soil layer (cm)	Field capacity (cm^3^·cm^-3^)	Saturated capacity (cm^3^·cm^-3^)	Wilting point (cm^3^·cm^-3^)	Bulk density (g·cm^-3^)
0-10	0.275	0.318	0.06	1.521
10-20	0.298	0.320	0.06	1.456
20-40	0.301	0.337	0.06	1.527

### 3.2 Experimental design

Due to regional field irrigation demands and agricultural input costs, the primary irrigation methods for alfalfa in the NIR include flood irrigation with Yellow River water, underground drip irrigation, and surface micro-spray irrigation. This study designed under irrigation by varied water and nitrogen levels based on annual average precipitation and alfalfa’s water and fertilizer requirements (Hu et al., 2019). Field experiments were conducted over four years (2017, 2018, 2022, 2023) under different irrigation methods (**Table 2**).

**Table 2:** The growth stages and irrigation treatments of alfalfa in 2017, 2018, 2022, and 2023.

Cuts time	Growth stage	Year	Division date of growth stages	Irrigation date	Irrigation amount (mm)			
					W1	W2	W3	W4
First cuts	Vegetative (Turn-green or Emergence)	2-yr-old 3-yr-old 1-yr-old	2017/3/28-4/22 2018/4/7-5/2 2023/5/1-5/20	4/2 4/20 -	52 52 -	60 60 -	67 67 -	67 67 -
	Branch	2-yr-old 3-yr-old 1-yr-old	2017/4/23-5/19 2018/5/3-5/21 2023/5/20-6/29	4/23 5/3 5/31	45 45 60	52 52 -	52 52 -	67 67 -
	Branch	2-yr-old 3-yr-old 1-yr-old	2017/4/23-5/19 2018/5/3-5/21 2023/5/20-6/29	5/12 5/14 6/9(6/22)	37 37 60	45 45 -	52 52 -	52 52 -
	Early flowering	2-yr-old 3-yr-old 1-yr-old	2017/5/20-5/24 2018/5/22-6/1 2023/6/30-7/14	5/20 5/22 7/9	37 37 60	37 37 -	45 45 -	52 52 -
Second cuts	Vegetative	2-yr-old 3-yr-old 1-yr-old 1-yr-old	2017/5/25-6/8 2018/6/2-6/22 2022/7/8-7/18 2023/7/15-7/26	6/3 6/12 7/8 7/22	45 45 45 60	52 52 52 -	52 52 65 -	60 60 60 -
	Branch	2-yr-old 3-yr-old 1-yr-old 1-yr-old	2017/6/9-6/20 2018/6/23-7/5 2022/7/19-7/28 2023/7/27-8/5	6/9 6/24 7/19 -	37 37 37 -	45 45 52 -	52 52 45 -	52 52 52 -
	Early flowering	2-yr-old 3-yr-old 1-yr-old 1-yr-old	2017/6/21-6/27 2018/7/6-7/8 2022/7/29-8/10 2023/8/6-8/23	6/22 7/6 7/29 8/6	37 37 37 60	37 37 45 -	45 45 64 -	52 52 52 -
Third cuts	Vegetative	2-yr-old 3-yr-old 1-yr-old	2017/6/28-7/13 2018/7/9-7/29 2022/8/11-9/1	7/6 7/16 -	45 45 -	52 52 -	52 52 -	60 60 -
	Branch	2-yr-old 3-yr-old 1-yr-old	2017/7/14-7/23 2018/7/30-8/6 2022/9/2-9/23	7/14 7/30 8/11	37 37 37	37 37 45	45 45 52	52 52 60
	Early flowering	2-yr-old 3-yr-old 1-yr-old	2017/7/24-7/31 2018/8/7-8/12 2022/9/24-10/4	7/24 8/7 9/2	30 30 45	37 37 52	45 45 46	52 52 60
Fourth cuts	Vegetative	2-yr-old 3-yr-old	2017/8/1-8/6 2018/8/13-8/18	–	- -	- -	- -	- -
	Branch	2-yr-old 3-yr-old	2017/8/7-9/6 2018/8/19-9/6	8/8 8/15	37 37	45 45	52 52	60 60
	Early flowering	2-yr-old 3-yr-old	2017/9/7-9/17 2018/9/7-9/29	9/4 9/7	37 37	45 45	52 52	60 60
Total		2017, 2018 2022 2023			476 200 360	544 246	611 260	679 269

1. During 2017-2018, the experimental design included various water and nitrogen treatments across a large plot area. Due to limitations in the daily irrigation capacity of the equipment, completing an irrigation plan typically took 2-3 days. As a result, irrigation dates for different treatments occasionally experienced delays of 1-3 days compared to the original schedule. 2. The irrigation scheduled for June 3, 2017, was canceled due to heavy rainfall of 27.0 mm prior to the planned irrigation.

The alfalfa varieties utilized in the experiments are Magnum 7 (planted on May 16, 2016) and Magnum 401 (planted on April 19, 2022, and May 1, 2023). Seeding was performed manually at a depth of 2 cm, with a seeding rate of 22.5 kg/hm^2^ and a row spacing of 15 cm. In the NIR, the optimal harvesting time for forage is during the early flowering stage (10% flowering), typically harvesting four cuts per year. However, in the first planting year, it usually harvests three cuts.

(1) In 2017 and 2018, underground drip irrigation was employed, with drip irrigation belts spaced 60 cm apart within each plot. The main pipeline was buried at a depth of 1.5 meters, and capillary pipes were placed 20 cm deep. Each plot was equipped with independent valves for irrigation control, and water application was regulated using a water meter. The experiment utilized a split-plot design, with primary treatments focusing on irrigation amounts and secondary treatments on nitrogen application rates. Irrigation levels were set at four amounts: 476 mm (W1), 544 mm (W2), 611 mm (W3), and 679 mm (W4). Nitrogen was applied at four rates: N0 (0 kg/hm^2^), N1 (60 kg/hm^2^), N2 (120 kg/hm^2^), and N3 (180 kg/hm^2^). Urea, containing 46.4% nitrogen, was applied concurrently with the first irrigation and after each cut.

(2) In 2022, surface micro-sprinkler irrigation was employed. Different irrigation amounts were designed for the second and third cuts of alfalfa, set at four levels: 200 mm (W1), 246 mm (W2), 260 mm (W3), and 269 mm (W4). No specific nitrogen application rate was assigned.

(3) In 2023, flood irrigation with Yellow River water was used. According to the traditional irrigation schedule of NIR, the irrigation amount for the first and second cuts of alfalfa was set at 360 mm (W1). No specific nitrogen application rate was assigned.

### 3.3 Data acquisition

#### 3.3.1 Shallow soil water content data acquisition

**Figure 2:**
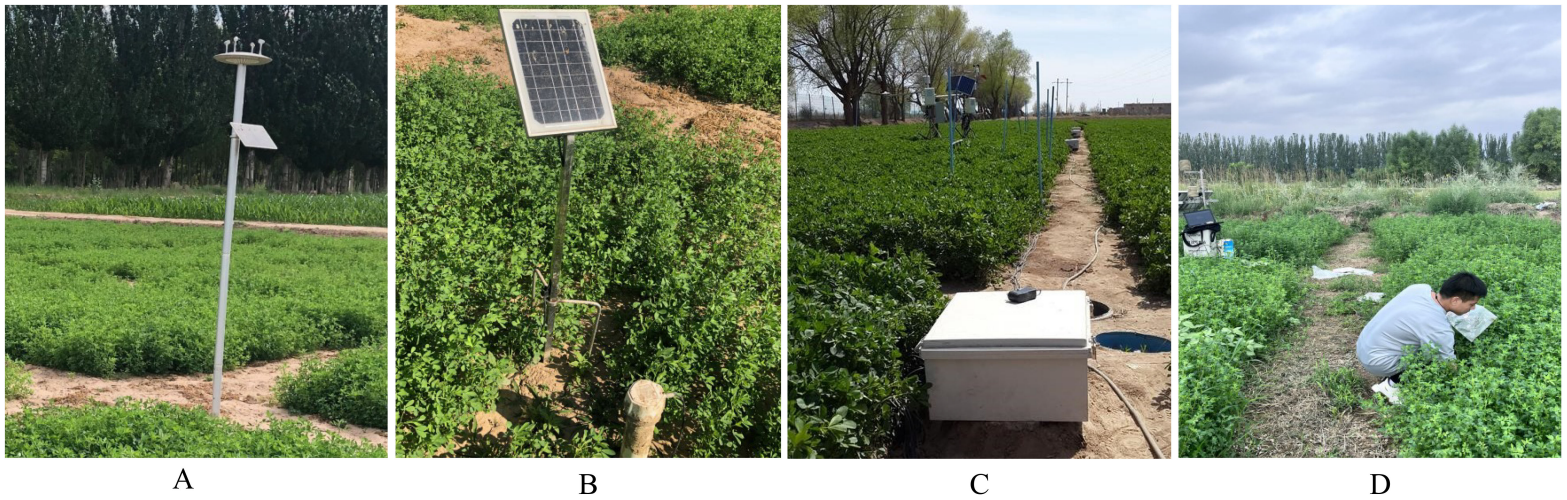
Data collection equipment and collection of crop indicators. **(A)** Weather Station; **(B)** Shang Crop & Soil Monitor; **(C)** MP406 Soil Monitor; **(D)** Experimental scenario.

This study utilized soil moisture sensors to collect soil water content and soil temperature data. The MP406 soil moisture sensor from Australia was used during 2017-2018 (**Figure 2C**), and from 2022 to 2023, the Shang Crop & Soil Monitor (INSENTEK, Hangzhou, China) was employed (**Figure 2B**).

Before deployment, we conducted laboratory calibration on various devices, achieving sensor accuracies ranging from ±1% to ±2%. These sensors measure soil water content (SWC) (%) and soil temperature (°C) across different soil layers (0-10 cm, 10-20 cm, and 20-30 cm). Data collection intervals were 30 minutes from 2017 to 2018 and 10 minutes from 2022 to 2023, enabling 24-hour real-time monitoring. Due to experimental constraints, SWC was measured across seven different water and nitrogen treatments during 2017-2018. This included various nitrogen treatments (W2N0, W2N1, W2N2, W2N3) under consistent water levels and different water treatments (W1N2, W3N2, W4N2) under uniform nitrogen levels.

#### 3.3.2 Meteorological data acquisition

This study utilized the Tianqi meteorological monitoring station (INSENTEK, Hangzhou, China) to gathered environmental data (**Figure 2A**). The station primarily collected environmental temperature (°C), humidity (% RH), solar radiation (MJ/m^2^), wind speed (m/s), and rainfall (mm). Data collection intervals were 30 minutes from 2017 to 2018 and 10 minutes from 2022 to 2023, enabling 24-hour real-time monitoring. A total of 49,358 meteorological data points were collected during the growth period of alfalfa over the past four years. The average monthly temperature recorded in this dataset is approximately 20°C. Rainfall mainly comprised light precipitation, with occasional heavy rain or rainstorms occurring primarily in July and August each year.

#### 3.3.3 Alfalfa data acquisition

This study collected comprehensive crop data, including alfalfa growth stage, leaf area index (LAI), yield, and harvesting time (**Figure 2D**). When alfalfa entered the turn-green (or vegetative) stage, three random sample plants per plot were selected weekly for height measurement, leaf area, and LAI calculation (Gower et al., 1999), adjusting for rain delays. At the early flowering stage, uniform alfalfa plants were selected for cutting. Using the diagonal method, three 1 m × 1 m quadrats per plot were randomly selected, maintaining a 5 cm stubble height. After weed removal, fresh yield was measured, and approximately 300 g of fresh alfalfa samples were air-dried in the laboratory to a constant mass for hay yield determination. Growth and harvesting times of each cut were observed and recorded in the field.

## 4 Description and analysis of dataset

This shallow soil moisture dataset for alfalfa consists of six parts, including growth stage data,leaf area index data, meteorological data, soil temperature data, soil water content data, and yield data. To facilitate data usage, we have annotated the file names in the dataset. This collection comprises 139,213 alfalfa soil moisture measurements at various depths across different water levels. Specific details of the dataset are provided in **Table 3**.

**Table 3:** Summary of six parts of the dataset.

Dataset	Folder name	Data indicators	Sample size
Soil water content data	2017 data	0-10 cm, 10-20 cm, and 20-30 cm	31206
	2018 data	0-10 cm, 10-20 cm, and 20-30 cm	36339
	2022 data	0-10 cm, 10-20 cm, and 20-30 cm	50868
	2023 data	0-10 cm, 10-20 cm, and 20-30 cm	20800
Soil temperature data	ST data	0-10 cm, 10-20 cm, and 20-30 cm	81564
Meteorological data	Meteorological data	Temperature	49358
		Dew point temperature	
		Humidity	
		CO_2_ concentration	
		Wind speed	
		Solar radiation	
		Rainfall	
Leaf area index data	LAI data	Leaf area index	695
		Plant height	
		Stem diameter	
Growth stage data	Growth stage data	Growth stage date	46
Yield data	Yield data	Yield	37

**Figure 3:**
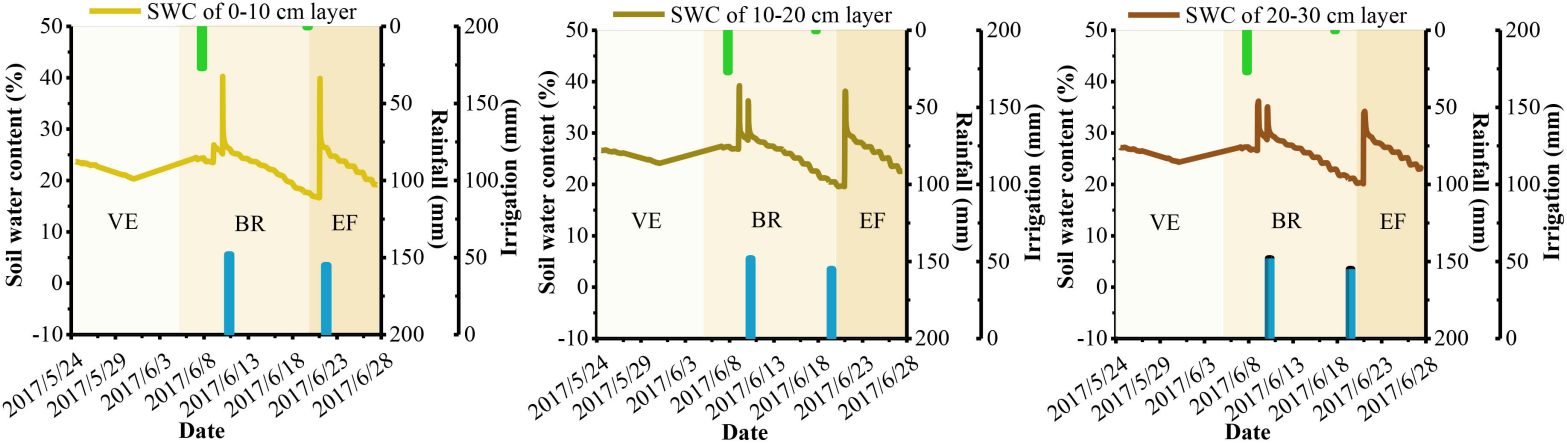
Soil water content at shallow depths of alfalfa under W2N3 treatment of the second cuts in 2017. VE is the vegetative (or turn-green) stage. BR is the branch stage. EF is to early flowering stage. The same is below.

**Figure 4:**
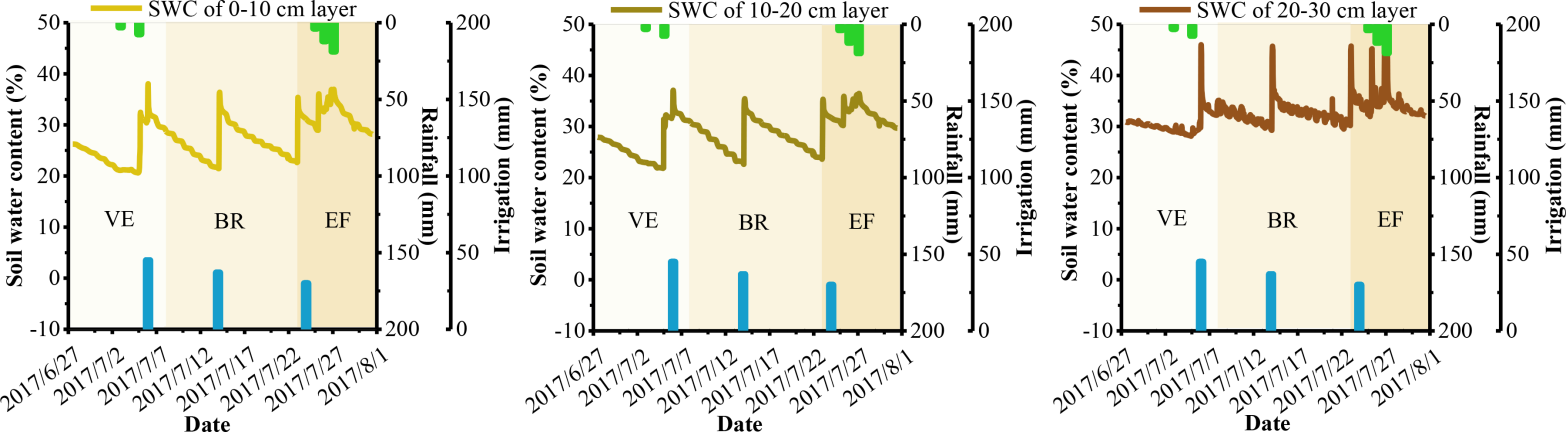
Soil water content at shallow depths of alfalfa under W1N2 treatment of the third cuts in 2017.

**Figure 5:**
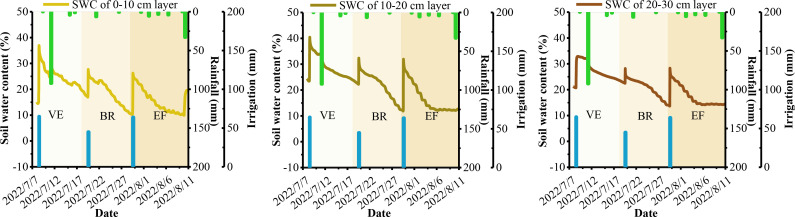
Soil water content at shallow depths of alfalfa under W3 treatment of the second cuts in 2022.

**Figure 6:**
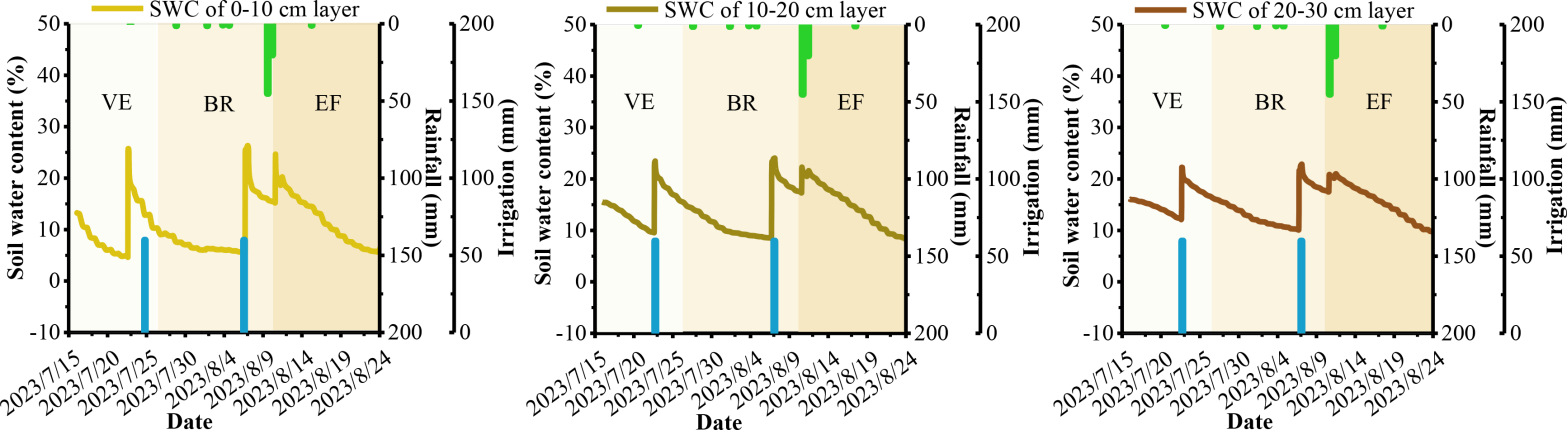
Soil water content at shallow depths of alfalfa under W1 treatment of the second cuts i`n 2023.

Soil moisture plays a crucial role in the atmospheric dynamics-alfalfa-soil system and is essential for researching alfalfa growth, root nutrition, yield, and variety formation. Soil basal determines the upper limit of SWC at various depths, while meteorological conditions, crop growth, soil basal, and field management practices drive dynamic changes in soil moisture across different growth periods. **Figures 3**–**6** show the dynamic trends of soil moisture at multiple depths during various precipitation years, cuts, and differing water and nitrogen levels selected from the dataset.

**Figures 3**–**6** show that the overall trend of soil moisture changes at multiple depths in alfalfa aligns with the expected soil moisture range for each soil layer. During significant precipitation or irrigation events, the SWC is high, often reaching saturation at different depths. Conversely, during light precipitation, low irrigation, or when SWC approaches the wilting point, the SWC generally reaches the soil’s field capacity. Notably, under light precipitation conditions, shallow soil moisture trends to increases, while deeper soil moisture often decrease. The figures indicate that the SWC at depths of 0-10 cm and 10-20 cm is significantly influenced by solar radiation and evapotranspiration. The SWC in these upper layers tends to be lower than in the 20-30 cm layer, aligning with findings from Yu et al. (2020) and Ait Hssaine et al. (2020). The small difference in SWC between the 0-10 cm and 10-20 cm layers may be attributed to similar environmental factors, as noted by Liu et al. (2019). Therefore, since rainfall primarily consists of light precipitation in the NIR, the analysis and prediction of shallow soil moisture are crucial for the effective utilization of micro-rainfall in the region. Additionally, the experimental data indicate that the W2 treatment is a more suitable water configuration for alfalfa under underground drip irrigation, while the W3 treatment is better suited for surface micro-spray irrigation.

Additionally, a comprehensive analysis of the dataset found that within the same precipitation year, low nitrogen levels had a relatively small effect on soil moisture compared to other nitrogen levels and did not significantly impact alfalfa growth. In different planting years, varying nitrogen application rates influenced alfalfa growth differently, even under the same irrigation amounts. Regarding nitrogen application, recommendations vary among studies. Some researchers suggest that timely nitrogen application can increases yield when soil NO_3_^-^ content is below 15 mg/kg (Hannaway and Shuler, 1993). Conversely, excessive nitrogen application may inhibit rhizobia development in alfalfa, thereby reducing nitrogen fixation and economic benefits (Markus and Battle, 1965; Hojjati et al., 1978). Thus, nitrogen application for alfalfa is complex and needs to consider factors such as grass age, soil basal, economic costs, and environmental conditions. The dataset from this study includes shallow soil moisture data for alfalfa with and without nitrogen application, providing a valuable reference for future research. However, the dataset focuses specifically on soil moisture within the shallow root concentration area of alfalfa in irrigated areas. Future research will concentrate on the following aspects: 1) conducting more extensive field experiments to collect deep soil moisture data of alfalfa, particularly tracking changes under dry farming conditions; 2) introducing deep learning methods to enhance the efficacy of precision irrigation management and prediction in alfalfa fields.

## 5 Potential use

With the rapid development of deep learning, the dataset established in this study has significant practical implications for predicting soil moisture at shallow depths in alfalfa within the NIR. Researchers can use this dataset to integrate complex environment information, including meteorology, crop growth, and field management, to develop a shallow soil moisture prediction model for alfalfa. Furthermore, a deep learning model developed using this dataset can be combined with the alfalfa model, providing crucial data support for yield estimation and the precise management of water and nitrogen in the field.

## Data Availability

The datasets presented in this study can be found in online repositories. The names of the repository/repositories and accession number(s) can be found below: https://github.com/geyongqi/SM-NIR.
